# Identification and characterisation of vaginal lactobacilli from South African women

**DOI:** 10.1186/1471-2334-13-43

**Published:** 2013-01-26

**Authors:** Sonal Pendharkar, Tebogo Magopane, Per-Göran Larsson, Guy de Bruyn, Glenda E Gray, Lennart Hammarström, Harold Marcotte

**Affiliations:** 1 Division of Clinical Immunology, Department of Laboratory Medicine, Karolinska University Hospital, Huddinge, Stockholm, Sweden; 2 Perinatal HIV Research Unit (PHRU), Chris Hani Baragwanath Hospital, Soweto, Johannesburg, South Africa; 3 Department of Obstetrics and Gynaecology Kärnsjukhuset, Skaraborg hospital and University of Skövde, Skövde, SE-541 85, Sweden; 4 Department of Laboratory Medicine, Division of Clinical Immunology, F79, Karolinska Institutet, Stockholm, S-141 86, Sweden

**Keywords:** Bacterial vaginosis, *Lactobacillus*, South Africa, Hydrogen peroxide

## Abstract

**Background:**

Bacterial vaginosis (BV), which is highly prevalent in the African population, is one of the most common vaginal syndromes affecting women in their reproductive age placing them at increased risk for sexually transmitted diseases including infection by human immunodeficiency virus-1. The vaginal microbiota of a healthy woman is often dominated by the species belonging to the genus *Lactobacillus* namely *L. crispatus, L. gasseri, L. jensenii* and *L. iners,* which have been extensively studied in European populations, albeit less so in South African women. In this study, we have therefore identified the vaginal *Lactobacillus* species in a group of 40 African women from Soweto, a township on the outskirts of Johannesburg, South Africa.

**Methods:**

Identification was done by cultivating the lactobacilli on Rogosa agar, de Man-Rogosa-Sharpe (MRS) and Blood agar plates with 5% horse blood followed by sequencing of the 16S ribosomal DNA. BV was diagnosed on the basis of Nugent scores. Since some of the previous studies have shown that the lack of vaginal hydrogen peroxide (H_2_O_2_) producing lactobacilli is associated with bacterial vaginosis, the *Lactobacillus* isolates were also characterised for their production of H_2_O_2_.

**Results:**

Cultivable *Lactobacillus* species were identified in 19 out of 21 women without BV, in three out of five women with intermediate microbiota and in eight out of 14 women with BV. We observed that *L. crispatus, L. iners, L. jensenii, L. gasseri* and *L. vaginalis* were the predominant species. The presence of *L. crispatus* was associated with normal vaginal microbiota (P = 0.024). High level of H_2_O_2_ producing lactobacilli were more often isolated from women with normal microbiota than from the women with BV, although not to a statistically significant degree (P = 0.064).

**Conclusion:**

The vaginal *Lactobacillus* species isolated from the cohort of South African women are similar to those identified in European populations. In accordance with the other published studies, *L. crispatus* is related to a normal vaginal microbiota. Hydrogen peroxide production was not significantly associated to the BV status which could be attributed to the limited number of samples or to other antimicrobial factors that might be involved.

## Background

The vaginal microbiota of a healthy woman is often dominated by lactobacilli. The most commonly identified *Lactobacillus* species include *L. crispatus, L. gasseri, L. jensenii* and *L. iners*[1-3]. Lactobacilli are thought to play an important role in protecting the host from urogenital infection by lowering the environmental pH through lactic acid production, by producing various bacteriostatic and bactericidal substances such as hydrogen peroxide (H_2_O_2_) and through competitive exclusion. Decrease in the number of lactobacilli and overgrowth of diverse anaerobes is associated with an increased incidence of bacterial vaginosis (BV) [4,5], which is one of the most common vaginal syndromes among women in their reproductive age [6-8]. BV is associated with an increased susceptibility to sexually transmitted diseases including infection by human immunodeficiency virus-1 (HIV-1) [9-11] where the elevated vaginal pH associated with BV may allow the survival of HIV. Moreover, BV associated microbiota has been shown to enhance HIV-1 transcription and replication [12].

It has previously been shown that exogenously applied lactobacilli can persist on the mucosal surface of the vagina, displace BV and restore a normal vaginal microbiota [13,14]. Given the high prevalence of BV in women in many sub-Saharan African countries with HIV epidemics (20%-60%), colonisation by exogenous lactobacilli may help to reduce the risk of HIV-1 transmission [9]. Furthermore, delivery of a microbicide by lactobacilli colonising the vagina has previously been suggested as a strategy for prevention of HIV transmission, particularly in developing countries [15].

Hence, a better understanding of the species composition and ecology of bacterial ecosystems may help to develop better prophylaxis against BV and HIV. If the *Lactobacillus* species isolated from African women are similar to those in America and Europe, where probiotic studies have been performed, similar strategies utilising probiotic and engineered lactobacilli could be applied in Africa. Some studies reported differences in the composition of vaginal microbiota among different ethnic groups in North America, where a higher incidence of vaginal communities not dominated by lactobacilli was observed in black women [16,17]. However, the few studies performed in Africa to date suggest that the vaginal *Lactobacillus* species predominant among black women are similar to those dominating in American and European studies [18,19].

In preparation to study the colonisation efficiency of exogenously applied vaginal lactobacilli and to treat BV in South African women, we have therefore identified the cultivable vaginal *Lactobacillus* species in women with or without BV in Soweto.

## Methods

### Sample collection

Forty premenopausal and HIV uninfected black women aged 18–44 years with or without BV were recruited for the study. Study participants were randomly selected among women who visited the Perinatal HIV Research Unit (PHRU) in Chris Hani Baragwanath Hospital, Soweto and received voluntary HIV counseling and testing. Inclusion criteria were their willingness to provide the informed consent, passing the assessment of understanding, BHCG negative status (non-pregnant) and belonging to a low risk group for HIV acquisition. The women were non-menstruating and not taking antibiotics at the time of sample collection. Vaginal swabs were collected in Amies agar gel medium with charcoal (Copan Venturi Transsystem® Copan Diagnostics, Italy). Simultaneously, a second similar swab was used to prepare a smear on a glass slide for BV scoring. The ethical approval for collection of swab samples was granted by Human Research Ethics Committee (Medical), University of the Witwatersrand, Johannesburg (Ethical permit number M011138). A regional ethical permit was also granted by Regionala Etikprövningdnämnden, Stockholm, for analysing these samples (Diarienummer: 2012/1362-31/1).

### Cultivation of lactobacilli

The swabs were transported at room temperature to the laboratory in Sweden within a maximum of two days. The swabs were directly streaked onto Rogosa agar (BD Difco™ Rogosa SL agar, Becton, Dickinson and Company, Spark, MD), Columbia agar (BD Difco™ Columbia Blood Agar Base) with 5% horse blood and de Man-Rogosa-Sharpe (MRS) agar (BD Difco™ Lactobacilli MRS broth) within 24 hours of arrival. The swabs were then vortexed in 1ml sterile PBS (pH 7.4) to prepare bacterial suspensions. The bacterial suspensions were diluted tenfold serially (three dilutions per suspension) and each dilution (100 μl) was plated onto the three different agar plates as described above [20]. Plates were incubated for 48 hours at 37°C in an incubator in anaerobic condition using BD GasPack™ EZ gaz generating systems (Becton, Dickinson and Company, Sparks, MD). Colonies with different morphology yielding variable rods were re-streaked. Respective colonies from re-streaked plates were Gram stained and the colonies with Gram positive rods were used to inoculate the MRS broth medium. Small transparent colonies from blood agar plates yielding Gram positive bacilli not growing in MRS broth were collected directly from the plates and used for genomic DNA isolation. Glycerol stocks (15%) were prepared and stored at −80°C. Eight to 12 different colonies were picked per sample.

### Identification of lactobacilli

Genomic DNA was extracted from lactobacilli using Qiagen’s DNAeasy Blood & Tissue extraction kit (Qiagen GmbH, Hilden, Germany). The isolates were identified to the species level by sequencing the 16S ribosomal RNA (rRNA) gene as described previously [21]. Briefly, the complete 16S rRNA gene (1.5 kb) was amplified by PCR using the primers P0 (5^′^-GAGAGTTTGATCCTGGCTCAG-3^′^) and P6 (5^′^-CTACGGCTACCTTGTTACGA-3^′^) and sequenced. The obtained sequences were then subjected to nucleotide-nucleotide BLAST using BLASTN ( http://www.ncbi.nlm.nih.gov/) and subsequently compared to the 16SrRNA sequences of typed strains to validate the results. The identification of *Lactobacillus* species was considered confirmed if the sequences showed 99-100% homology with the typed strain. The following typed strains and 16S rRNA gene sequence (Genbank accession number) were used for the comparisons: *L. mucosae* strain CCUG 43179 (AF126738), *L. paracasei* strain ATCC 25302 (HQ423165), *L. ruminis* strain ATCC 27782 (CP003032) and NBRC 102161 (AB326354), *L. crispatus* strain ATCC 33820 (AF257097) & DSM 20584T (FR683088), *L. coleohominis* DSM 14060 (AM113776), *L. vaginalis* strain ATCC 49540 (AF243177) and KC19 (AF243154), *L. iners* strain DSM 13335 (NR_036982) and CCUG 28746T (Y16329), *L. gasseri* ATCC 33323 (AF519171) and *L. jensenii* strain ATCC 25258 (AF243176).

### Determination of BV

Vaginal smears were Gram stained and graded on a 10 point scale based on the presence of lactobacilli and other anaerobes as described by Nugent [22] for normal microbiota (score 0–3), intermediate microbiota (score 4–6) and BV (score 7–10). In brief, a score of zero to four is given individually for the presence of lactobacilli and for *Gardnerella*/*Bacteroides* morphotypes and a score of zero to two for curved Gram variable rods. The sum of all scores is a representative of the BV score. The presence of epithelial cells covered with bacteria (clue cells), a critical component of the Amsel’s clinical criteria for BV, was also noted [23].

### Hydrogen peroxide (H_2_O_2_) production test

*Lactobacillus* isolates were streaked onto a 20 ml MRS agar plate containing 0.25 mg/ml 3,3’, 5,5’-tetramethylbenzidine (TMB) (Sigma-Aldrich, St-Louis, MO) and 0.01 mg/ml of horseradish peroxidase (HRP) (Sigma-Aldrich). Plates were incubated in anaerobic condition using the BD Gas Pack™ anaerobic container system for 72 hours and were exposed to air for 30 minutes before scoring them for blue coloration. HRP generates O_2_ from any H_2_O_2_ produced by the lactobacilli which in turn oxidises the TMB substrate to form a blue pigment. On the basis of the blue coloration, isolates were scored for H_2_O_2_ production as 0 for no blue coloration, 1 for light blue, 2 for moderate and 3 for dark blue coloration. Since it was difficult to culture *L. iners* on MRS agar medium, *L.* iners isolates were not included in the H_2_O_2_ production test.

### Statistical analysis

Fisher’s exact test was performed to test the relation between BV status (normal microbiota vs. BV) and the presence of any lactobacilli*,* specific *Lactobacillus* species and high/low H_2_O_2_-producing lactobacilli. Two-tailed P values less than 0.05 were considered statistically significant. All the comparisons were performed with GraphPad Prism 4 software (GraphPad Software, Inc., La Jolla, Ca).

## Results

### BV status and *Lactobacillus* colonisation

Nugent scoring of the Gram stained vaginal smears revealed that 21 women had a normal vaginal microbiota, five had an intermediate microbiota and 14 had BV. The presence of clue cells was observed in all the women with BV but not in women with normal or intermediate microbiota (Table 1). *Lactobacillus* colonisation was observed in women both with and without BV, where cultivable *Lactobacillus* species were identified in 19 out of 21 women with normal microbiota, in three out of five women with intermediate microbiota and in eight out of 14 women with BV.

**Table 1:** Study participants characteristics

	**Normal**	**Intermediate**	**BV**	**Women Total**
	**N=21**	**N=5**	**N=14**	**40**
Mean age (range)	27 (19–40)	29 (22–40)	27 (19–44)	27 (18–44)
Median (and range) of Nugent scores	1 (0–2)	5 (4–6)	8 (8–10)	
Number of samples with clue cells	0	0	14	14
Presence of *Gardnerella* morphotypes on microscopy	0	5	14	19
Presence of *Mobiluncus* morphotypes on microscopy	0	0	3	3
Presence of any *Lactobacillus* species on culture	19	3	8	30
Presence of *L. crispatus* on culture	10	0	0	10

**Figure 1:**
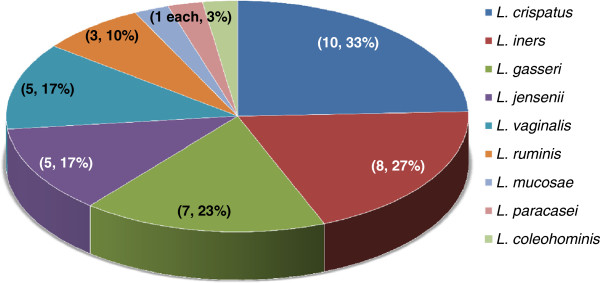
**Distribution of colonising *Lactobacillus *species in South African women.** The number and percentage of women colonised by the *Lactobacillus* species is indicated in parenthesis.

**Figure 2:**
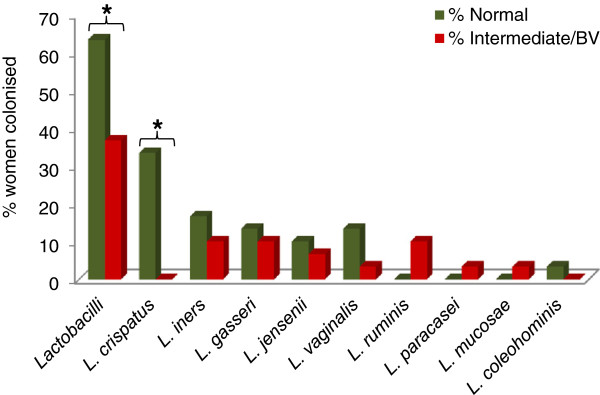
**Association between colonising lactobacilli and BV status defined by Nugent Score.** * Significantly different (p value < 0.05) according to a two tailed Fisher’s exact test.

A total of 196 isolates were cultured from 40 swabs and 30 women (75%) were identified with cultivable *Lactobacillus* species. *L. crispatus*, *L. iners and L. gasseri* were identified in 10 (33%), 8 (27%) and 7 (23%) women respectively, whereas *L. vaginalis* and *L. jensenii* colonised five (17%) women each. These were the most commonly identified *Lactobacillus* species. Other *Lactobacillus* species identified were *L. ruminis, L. mucosae, L. paracasei* and *L. coleohominis* (Figure 1). Among the 30 women harbouring lactobacilli, nine were colonised by more than one *Lactobacillus* species (Table 2). *L. crispatus* was only isolated from women with normal microbiota whereas the other predominant *Lactobacillus* species (*L. jensenii*, *L. gasseri*, *L. vaginalis* and *L. iners*) were isolated from women both with and without BV (Figure 2). The two women who were colonised by *L. ruminis* only and the one woman who was colonized by a mix of *L. ruminis, L. mucosae* and *L. paracasei* were identified with BV and intermediate microbiota, respectively. Colonisation by any lactobacilli and in particular *L. crispatus* was significantly associated with a normal vaginal microbiota (Fisher test, normal vs. BV microbiota, P = 0.0386 and P = 0.024 respectively) but not the colonisation by other *Lactobacillus* species.

**Table 2:** **Colonising *Lactobacillus *species in thirty women**

**Colonising species**	**No. of women colonised**	**Nugent score**		
		**0-3**	**4-6**	**7+**
***L. crispatus***	4	4		
***L. iners***	7	4	1	2
***L. gasseri***	4	2	1	1
***L. jensenii***	2	1		1
***L. vaginalis***	2	1		1
***L. ruminis***	2			2
***L. crispatus, L. jensenii***	2	2		
***L. crispatus, L. gasseri***	1	1		
***L. crispatus, L. vaginalis***	1	1		
***L. crispatus, L. coleohominis***	1	1		
***L. iners, L. vaginalis***	1	1		
***L. crispatus, L. vaginalis, L. gasseri***	1	1		
***L. mucosae, L. paracasei, L. ruminis***	1		1	
***L. gasseri, L. jensenii***	1			1

### Hydrogen peroxide (H_2_O_2_) production and BV status

All the isolates but *L. iners* were tested and scored for H_2_O_2_ production on a scale of zero to three on the basis of blue coloration. *L. iners* was not evaluated for H_2_O_2_ production since they do not grow on MRS agar plates. Production of H_2_O_2_ was observed in the majority of the *L. crispatus* (90%), *L. jensenii* (86%) and *L. vaginalis* (80%) isolates and in a lower proportion of the *L. gasseri* isolates (30%). *L. ruminis, L. coleohominis, L. paracasei* were non-producers. Among the most commonly isolated species, the proportion of isolates producing high levels of H_2_O_2_ (score 2 and 3) was significantly higher (Fisher test, P<0.001) for *L. jensenii* (52 %), *L. crispatus* (33 %) and *L. vaginalis* (79%) than *L. gasseri* (0%).

**Figure 3:**
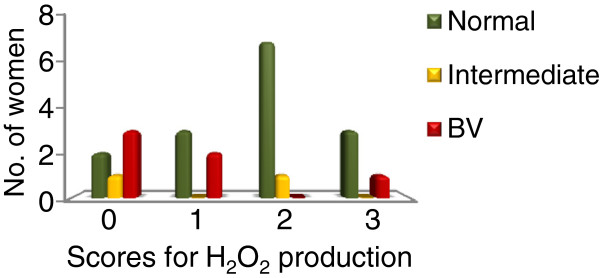
Relation between hydrogen peroxide production by lactobacilli and the Nugent score.

The isolation of strong H_2_O_2_ producers (either *L. jensenii*, *L. crispatus* or *L. vaginalis* with a score of 2 or 3) was in general more frequent in women with a normal microbiota than in the women with an intermediate microbiota or BV. However H_2_O_2_ production as an individual factor did not contribute significantly to the normal vaginal microbiota (Fisher test, normal Vs. BV microbiota, P = 0.064) (Figure 3).

## Discussion

With an increasing number of HIV-1 infected individuals and women being at a higher risk of acquiring HIV infection, it is of great importance to improve women’s vaginal health especially in the high risk population for sexually transmitted diseases. It is estimated that nearly one-third of all new HIV cases in South African population might be prevented if all cases of BV could be cured [9]. With an aim to restore the vaginal health through the administration of exogenous *Lactobacillus* among South African women, it was important to identify the *Lactobacillus* species that colonise women in this region. Our finding indicates that the *Lactobacillus* species (*L. crispatus, L. iners, L. gasseri, L. vaginalis and L. jensenii*) that dominate the vaginal microbiota in our cohort are similar to those predominating in European and American women [1,2,24]. Our results are in accordance with studies conducted by Akunam *et al*. [19] in Nigeria and more recently, Damelin *et al*. in South Africa [18], where the same predominant vaginal *Lactobacillus* species were isolated.

As previously reported, the presence of *Lactobacillus* species was a major determinant of a normal vaginal microbiota [25,26]. More particularly, the isolation of *L. crispatus* was strongly associated with the normal vaginal microbiota of women in the study as none of the women colonised by *L. crispatus* had BV or an intermediate microbiota. A number of previous studies have also shown an association between absence of BV and the presence of *L. crispatus*[1,2,24]. Furthermore, longitudinal analysis of the microbiota has shown that the presence of *L. crispatus* promotes stability of the vaginal microbiota [26]. The ability of *L. crispatus* to produce H_2_O_2_ has been suggested as an important factor in maintaining the normal vaginal mirobiota as most of the *L. crispatus* strains were found to be strong H_2_O_2_ producers [5]. On the contrary, *L. iners*, *L. gasseri* and *L. jensenii* were isolated from women with normal microbiota and BV. The presence of these *Lactobacillus* species in women with BV could be due to their poorer colonisation resistance, thereby allowing overgrowth of other bacteria or due to their better resistance for the environmental conditions associated with BV. Longitudinal studies in pregnant women have shown that the women harboring these *Lactobacillus* species, particularly *L. gasseri* and *L. iners*, are more susceptible to BV compared to the women colonised by *L. crispatus*[26]. It has also been suggested that *L. iners* may become a dominant part of the vaginal microbiota when the microbiota is in a transitional stage between abnormal and normal [27].

The vast majority of clinical studies have established an inverse association between BV and the occurrence of vaginal H_2_O_2_-producing *Lactobacillus* species. In a 2-year follow-up study, Hawes *et al*. documented that the acquisition of bacterial vaginosis was strongly associated with a lack or loss of hydrogen peroxide producing lactobacilli [5]. Although the results of the numerous clinical studies suggest that BV is caused by the lack of vaginal H_2_O_2_-producing *Lactobacillus* species; the established correlation may not necessarily indicate causality. Lactobacilli that produce H_2_O_2_ have been shown *in vitro* to inhibit the growth of various microorganisms, including *Gardnerella vaginalis*, anaerobes, *Neisseria gonorrhoeae* as well as the survival of HIV [28-31], but the relative contribution of the H_2_O_2_ produced by the *Lactobacillus* species to the overall antimicrobial effect is still a matter of debate. Under the hypoxic conditions that generally prevail in the vagina, H_2_O_2_ production by vaginal lactobacilli is undetectable (detection threshold 10 nM). Additionally, studies have shown that the cervicovaginal fluid and semen have a significant H_2_O_2_-blocking activity and that physiological concentrations of H_2_O_2_ below 100 μM did not kill any of the tested BV-associated bacteria [32].

In this study, we found that the majority of *L. crispatus, L. jensenii* and *L. vaginalis* isolates from South African women were producing H_2_O_2_, corroborating previous results [33,34]. Similar to the other reports, we have also observed that a high proportion of *L. crispatus*, *L. jensenii* and *L. vaginalis* isolates were strong H_2_O_2_ producers while all the *L. gasseri* isolates were low producers [24,30,33,35]. The presence of these strong H_2_O_2_ producers was higher in women with a normal microbiota although not to a significant level. The absence of significant inverse association between BV and the occurrence of vaginal H_2_O_2_-producing *Lactobacillus* species in the present study could be due to different factors. Our sample size was rather small and a single sampling occasion may not properly reflect the status of the vaginal microbiota of a woman as changes in the microflora during the menstrual cycle has been documented previously. Several studies have indicated that *Lactobacillus* growth increases throughout the menstrual cycle, but decreases during the menses [36,37]. Furthermore, we did not record any behavioral factors that might have affected the vaginal microbiota status during the study. Therefore, our results need to be confirmed in a larger cohort preferably using a longitudinal study design, combined with data on subjects' behaviors.

The production of H_2_O_2_ by *L. iners* isolates was not evaluated as these strains grow poorly on MRS containing TMB substrate. Most *L. iners* strains have been found to be non-H_2_O_2_ producers which might correlate with our finding that these species was isolated in both BV positive and negative women [33]. The production of H_2_O_2_ in this species should be assessed on medium adapted for the growth of *L. iners*. Furthermore, the proportion of H_2_O_2_ producing lactobacilli (and not only their presence) might also be important and the relative amount of lactobacilli should be determined by molecular methods. Finally, although the production of H_2_O_2_ by lactobacilli maybe an important factor for maintenance of the vaginal microbiota, other factors such as competition for adherence and production of other antimicrobial substances may also contribute to vaginal health and might act in synergy with H_2_O_2_. Comparative genomic studies of the lactobacilli isolated from the vagina of women with normal microbiota and BV might give a clue of the factors involved in colonisation resistance.

## Conclusions

The vaginal *Lactobacillus* species identified in black South African women from Soweto are similar to those identified in women from European populations. Colonising *Lactobacillus* species, in particular *L. crispatus,* contribute to a normal vaginal microbiota. High H_2_O_2_ producers were more frequently isolated from women without BV, although not to a statistically significant degree which might be due to the small sample size. The similarity in the species composition between European and African women suggest that, similar to the studies conducted in North European populations [12,21], administration of exogenously applied lactobacilli (probiotic or designer probiotics) in South African women could potentially be used for treatment of BV and thus reduce the occurrence of infection by sexually transmitted pathogens, including HIV-1.

## Abbreviations

BV: Bacterial Vaginosis; HIV-1: Human immunodeficiency virus-1; H2O2: Hydrogen peroxide; TMB: 3,3’, 5,5’-tetramethylbenzidine; HRP: Horseradish peroxidase.

## Competing interests

The authors declare that they have no competing interests.

## Authors’ contributions

GEG, GdB, HM, LH designed the research project, SP, TM and PL performed the experiments. Statistical analysis was performed by SP, HM and PG and the paper was written by SP, HM and LH. All authors read and approved the final manuscript.

## Pre-publication history

The pre-publication history for this paper can be accessed here:

http://www.biomedcentral.com/1471-2334/13/43/prepub
